# Synthesis of nanoscale CuO *via* precursor method and its application in the catalytic epoxidation of styrene

**DOI:** 10.1039/d1ra09384c

**Published:** 2022-02-18

**Authors:** Ruhul Amin Bepari, Palash Bharali, Birinchi Kumar Das

**Affiliations:** Department of Chemistry, Gauhati University Guwahati-781014 Assam India ruhulbepari@gmail.com birinchi.das@gmail.com; Department of Chemistry, B. N. College Dhubri-783324 Assam India; Department of Chemistry, Bhattadev University Bajali Pathsala-781325 Assam India

## Abstract

Nanoscale CuO with diameters in the range of 7–8 nm has been synthesized *via* a two-step precipitation–calcination method using copper(ii) isonicotinate tetrahydrate as the precursor. The first step involves the room temperature stirring of an alkaline ethanolic solution of the precursor which gives a non-crystalline CuO species, while the second step involves the calcination of the product of the first step at 180 °C to form nanocrystalline CuO which has been characterized by PXRD, TEM, SEM, H_2_-TPR and Raman spectroscopy, *etc.* The CuO material has shown excellent catalytic activity in the oxidation of styrene using TBHP as the oxidizing agent leading to complete styrene conversion with more than 95% styrene oxide selectivity at the end of 6 h. The oxide catalyst can be reused for at least 6 successive runs without significant loss in activity.

## Abstract 2

Nanoscale CuO with diameters in the range of 7–8 nm has been synthesized *via* a two-step precipitation–calcination method using copper(ii) isonicotinate tetrahydrate as the precursor.

## Graphical abstract

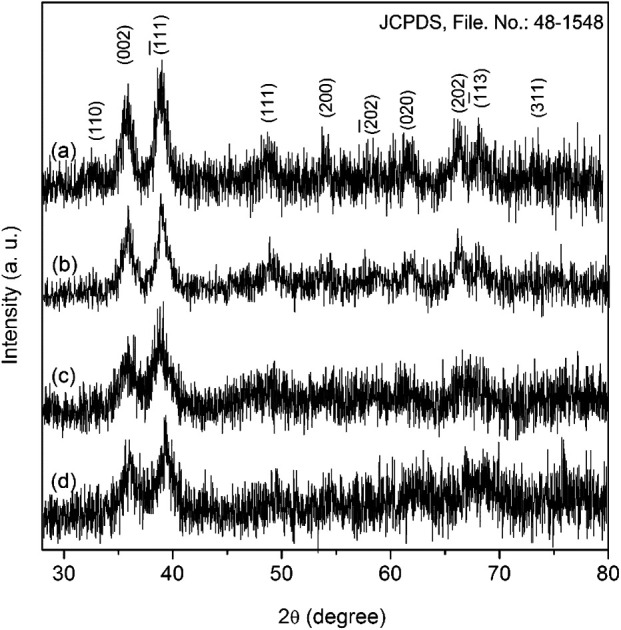

## 1 Introduction

Amongst the transition metals, copper finds numerous applications in diverse fields ranging from catalysis to drug delivery systems. Hence, currently researchers are exploring properties both of the elemental copper as well as its compounds for various applications. For example, the synergistic catalytic effect of the Se–Cu system has recently been used to produce α-ketoacetals by the activation of α-H of methyl ketones with molecular oxygen.^1^ In another instance, the synergistic catalytic activity of the Se–Cu catalyst has also been reported for the diethoxylation of halomethylene ketones using molecular oxygen as the green oxidant.^2^ Recently, Lei *et al.* have reported the high catalytic turnover numbers in the Buchwald–Hartwig coupling reaction of pyrimidin-2-amines over the polyaniline-supported copper catalyst (Cu@PANI) which was synthesized *via* the oxidative polymerization of aniline.^3^ Likewise, the oxides of copper, particularly the cupric oxide (CuO), have also attracted significant research interests in the recent years. This p-type semiconductor material with narrow band gap (1.7) eV finds important use in light emission,^4^ catalysis,^5^ gas-sensing,^6^ solar-energy harvesting,^6,7^ superconductivity and magnetoresistivity applications.^3,7^ Nanoscale CuO with various morphologies was successfully fabricated by several methods.^4–12^ In the recent times, researchers have been attracted towards the new trend of synthesizing copper oxide nanoparticles by adopting facile and green routes to fabricate this oxide with desired morphologies.^13–19^ In some instances, synthesis of CuO nanostructures *via* precursor method using different copper complexes as the precursors were also reported.^20,21^

The epoxidation of styrene, a terminal olefin, is of great significance because styrene oxide (SO) is an important organic intermediate for the synthesis of number of fine chemicals and pharmaceuticals. It is being traditionally produced by the epoxidation of styrene using stoichiometric amounts of peracids as the oxidizing agents.^22^ However, peracids are very expensive, hazardous to handle and non-selective for the epoxide formation. In order to overcome these shortcomings, metal oxide catalysts including both supported and unsupported oxides were explored in the epoxidation of this terminal olefin.^23,24^ Choudhary *et al.* reported the selective epoxidation of styrene over different alkaline earth or rare earth and transition metal oxides supported gold catalysts.^25–27^ The supported gold catalysts were found to be highly active for the epoxidation giving high epoxide selectivity with anhydrous TBHP as the oxidant. Supported CuO catalysts (*viz*. CuO/Si-MCM-41, CuO/Al_2_O_3_, CuO/Ga_2_O_3_ and CuO/In_2_O_3_) were also used for the epoxidation of styrene using TBHP as the oxidant.^28^ These authors reported a maximum styrene conversion of 74% with 78% epoxide selectivity over CuO/Ga_2_O_3_ catalyst. Chen *et al.* have found that 88.6% styrene conversion with 62.1% epoxide selectivity is possible *via* a 2 h epoxidation reaction over mesoporous silica shells coated CuO/CNCs catalyst under heterogeneous condition.^11^ In the recent past, works on the catalytic epoxidation of styrene using TBHP as the oxidant under heterogeneous condition have been reported.^29–35^ Although, some good works have been reported on the epoxidation of styrene over various metal oxide based catalysts under heterogeneous condition, in most of the cases, high styrene conversion with desired epoxide selectivity has been a challenging task.

Copper(ii) isonicotinate tetrahydrate, consisting of the hexacoordinate complex *trans*-[Cu(NC_5_H_4_-*p*-CO_2_)_2_(OH_2_)_4_], is a member of metal(ii) isonicotinate tetrahydrates which are hydrogen-bonded 3D solids characterized by poor solubility in water.^36^ This compound may be prepared in excellent yield by following the method developed earlier in this laboratory.^36^ Herein, we discuss results on the synthesis of CuO nanoparticles *via* a newly developed precipitation–calcination method using copper(ii) isonicotinate tetrahydrate as the precursor. Furthermore, catalytic performance of the synthesized oxide in the epoxidation of styrene using TBHP as the oxidant will also be discussed.

## 2 Experimental

### 2.1 Materials and methods

The reagents and solvents used in this work were obtained from commercial sources and utilized without further purification. Isonicotinic acid was obtained from Sigma-Aldrich (USA) while copper sulfate pentahydrate (CuSO_4_·5H_2_O) from Loba Chemie (India). Ethanol, sodium chloride and sodium hydroxide (NaOH) were purchased from E. Merck (India). Styrene and TBHP (aqueous weight 70%) were procured from Sigma-Aldrich (USA). A Philips X'pert Pro X-ray diffractometer with Cu-Kα radiation (*λ* = 1.5418 Å) was used to obtain the X-ray powder diffraction patterns with 2*θ* ranging from 25 to 80°. Transmission electron microscopy (TEM) images were recorded on two different high resolution transmission electron microscopes (HRTEM) *viz*. HRTEM, JEOL, model 1200 EX operating at 100 kV and TEM, JEM-2100, JEOL operating at 200 kV. FT-Raman spectra were recorded on a HoribaJobin-Yvon LabRAM HR Raman spectrometer using green light (514 nm) for the excitation. Temperature-programmed reduction (TPR) experiments were performed using a Micromeritics AutoChem 2910 analyzer equipped with a thermal conductivity detector (TCD). The materials were pretreated in a flow of pure argon at 250 °C for 1 h at a ramping rate of 5 °C. Then, H_2_-TPR experiments were carried out under the mixture of 5% H_2_ in Ar flowing (30 mL min^−1^) over 50 mg of the sample at a heating rate of 10 °C min^−1^. During catalytic reactions, the reaction mixture was analyzed and quantified by a Bruker 430 GC (FactorFour™: Capillary column, VF-1ms, 15 m, 0.25 mm, 0.25 μm) gas chromatographic technique. The products were identified by a PerkinElmer, Clarus 600 C Mass spectrometer equipped with an Elite 5 MS (non-polar) column: 30.0 m × 250 μm. The electrochemical studies were performed using an Autolabel electrochemical analyzer (Eco Chemie, The Netherlands) in a phosphate buffer saline (PBS, pH 7.0) containing 5 mM [Fe(CN)_6_]^3−/4−^as the redox probe.

### 2.2 Synthesis of CuO nanoparticles

Typically, 8.7 g of NaCl was taken into 75 mL of an alkaline ethanolic solution (0.1 M). To this solution 1.1 g of the copper complex, Cu(C_6_H_4_O_2_N)_2_·(H_2_O)_4_was added under constant mechanical stirring. The stirring was continued for 2 h at room temperature and the solvent was then removed at 85 °C on a water bath. The dried mass was ground finely on an agate mortar and finally calcined at 180 °C in a muffle furnace in static air for 3 h. The calcined powder was washed several times with water till the washings became chloride free (tested with AgNO_3_). The black CuO powder was dried at 60 °C for 6 h.

Formation and growth of the CuO nanoparticles were monitored in different stages of the reaction and under different reaction conditions. Thus, attempts were made to synthesize CuO particles with and without NaCl to examine whether NaCl has any effect on the size, morphology and surface defects of the particles. Moreover, after the room temperature stirring the intermediate product was subjected to oven drying for the preliminary characterization (XRD and Raman spectroscopy) to investigate whether the greenish Cu(OH)_2_ formed in the initial stage of the reaction transformed into CuO at room temperature condition or not. We will call the copper oxides as CuO-A (obtained by using NaCl and calcination), CuO-B (obtained by calcination without using NaCl), CuO-C (using NaCl and without calcination) and CuO-D (without using NaCl and without any calcination) in the further discussion (Table 1).

**Table 1:** Samples and reaction conditions under which they were prepared

Sample	Reaction conditions
CuO-A	Using NaCl and calcination
CuO-B	Calcination without using NaCl
CuO-C	Using NaCl and without calcination
CuO-D	Without calcination without using NaCl

### 2.3 Catalytic reaction

The catalytic reaction was carried out in a round bottomed flask fitted with a water-cooled condenser using 70% aqueous TBHP as the oxidant. An amount of 10 mg of the catalyst was added to the reaction mixture containing 5 mmol of styrene and 14 mmol of TBHP in 10 mL acetonitrile. The mixture was stirred constantly under reflux condition at 85 °C for different length of times. The progress of the reaction was monitored periodically by a GC. During the catalytic reaction, samples of the reaction mixture were withdrawn at regular time intervals and each time the catalyst was filtered off by centrifugation prior to the GC investigation. The products were identified by comparing them with authentic samples, and substrate conversion and product yields were determined from the peak areas.

## 3 Results and discussion

### 3.1 Synthesis and characterization

The present method developed by us has several advantageous features over the existing procedures for the fabrication of nanoscale CuO. Literature suggests that thermolytic solid-state reaction have traditionally been carried out to decompose copper complexes into CuO under robust condition.^20,21^ Such solid-state reactions are generally carried out at very high temperatures for long periods of time. As a result of this, large oxide particles with inhomogeneous size distributions are found to form. But, it is possible to obtain ultra-small copper oxide particles with uniform size distribution following our precipitation–calcination method. Although, the present method is very similar to the conventional sol–gel technique, it has some advantages over the latter as well. Unlike the sol–gel technique, where metal hydroxides are formed in the first step, the present method directly gives non-crystalline metal oxides in the first step (precipitation-step) and only a low-temperature heat treatment is then required to increase the crystallinity of the as-formed oxide particles. Additionally, the precursor species used in this method can easily be obtained under milder condition than those used in other methods.^20,37^ Furthermore, this method can easily be scaled-up on demand. It also provides the possibility to protect surface defects of the nanoparticles with the help of a no-toxic additive like NaCl.

**Fig. 1:**
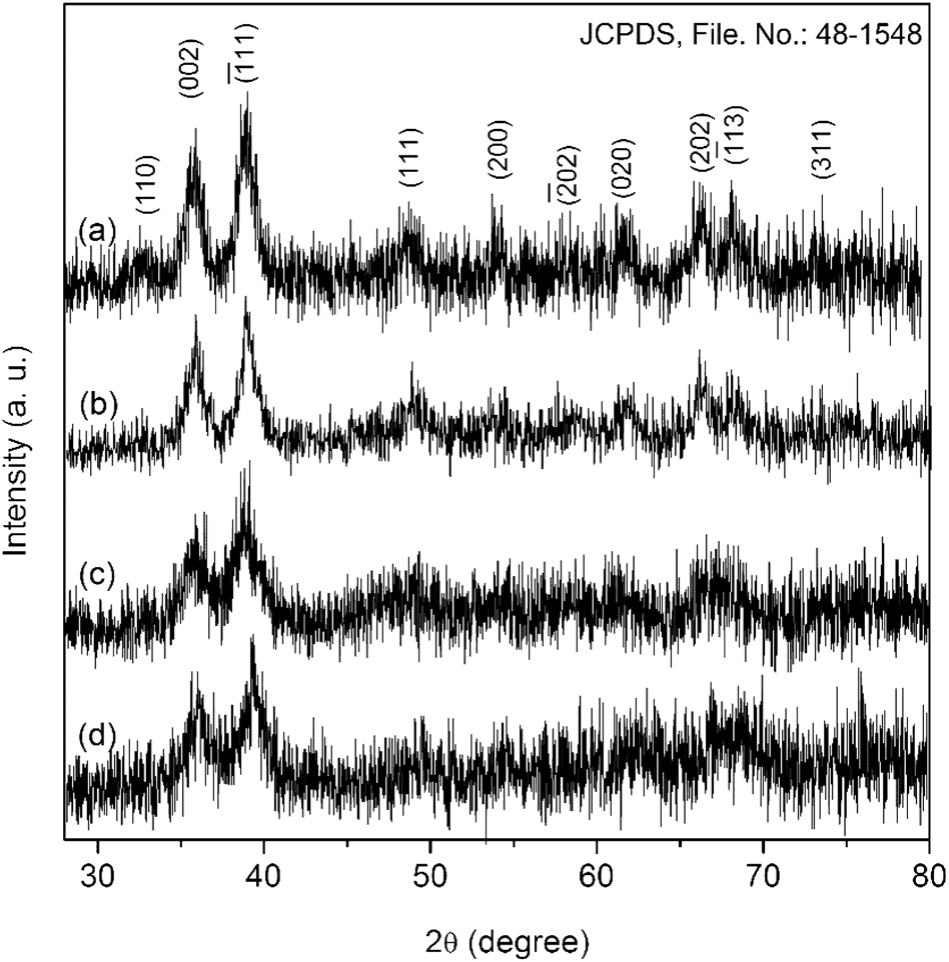
XRD patterns of (a) CuO-A, (b) CuO-B, (c) CuO-C and (d) CuO-D.

All the reflections in the diffractogram of CuO-A (Fig. 1a) can be indexed to pure monoclinic CuO with lattice constants *a* = 4.692 Å, *b* = 3.428 Å and *c* = 5.137 Å (JCPDS, ref. no. 48-1548). No characteristic peak for any impurity such as Cu(OH)_2_ or Cu_2_O can be detected. A comparison between the diffractograms of CuO-A and that of the other samples suggests that the sample CuO-B is also a phase-pure CuO. Three peaks appearing at the 2*θ* values of 35.9, 39.2, and 66.5° in the diffractograms of both CuO-C and CuO-D correspond to the typical(1̄11), (111) and (3̄11) reflections of CuO. Appearance of just three reflections suggests the non-crystalline nature of the materials. But heat treatment of these non-crystalline oxides (CuO-C and CuO-D) at 180 °C leads to the formation highly crystalline CuO-A and CuO-B respectively. Although, the CuO-A is the calcined-crystalline form of CuO-C and CuO-B is calcined-crystalline form of CuO-D, in the present work, all these four oxide samples were investigated separately in order to study the change in their microstructural properties and hence chemical activities. It is noteworthy that peak broadening due to the quantum confinement effect can clearly be observed in all the diffractograms.

**Fig. 2:**
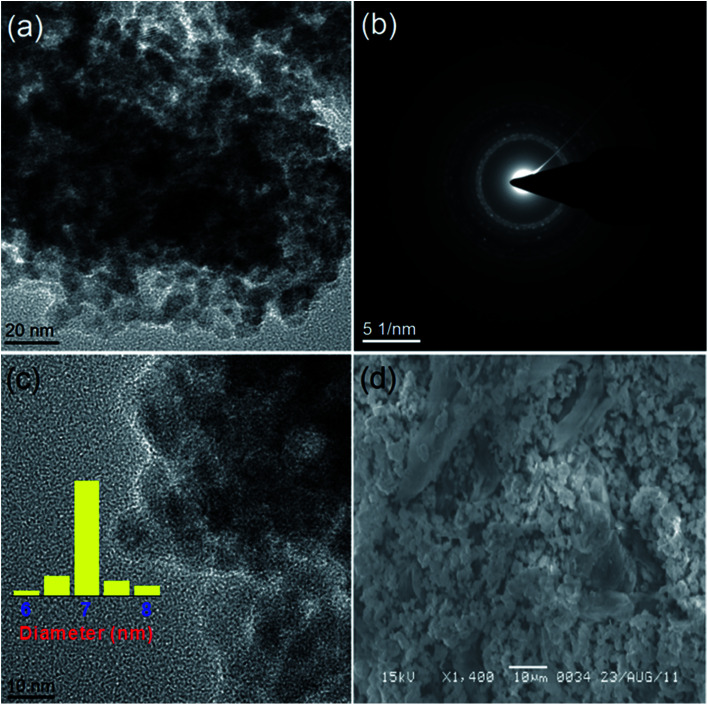
(a) TEM image, (b) SAED pattern, (c) HRTEM image and (d) SEM image of CuO-A.

**Fig. 3:**
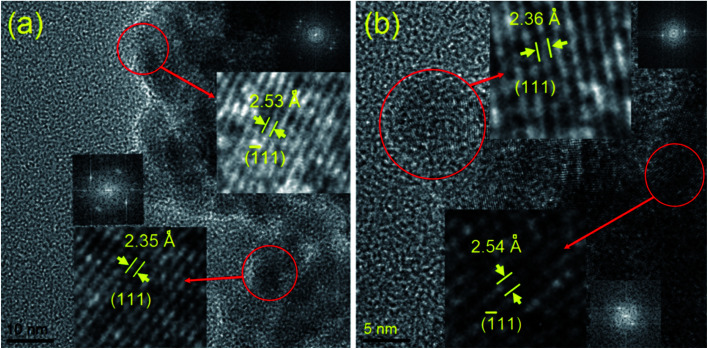
HRTEM image of (a) CuO-A and (b) CuO-B. Insets show their respective lattice spacings with corresponding FFT patterns.

The structure and morphology of all the synthesized samples were also studied further with the transmission electron microscopy (TEM) and scanning electron microscopy (SEM) techniques. The TEM and HRTEM images (Fig. 2 and 3) show the spherical oxide particles with an average diameter of 8 nm. Although, the particles are largely agglomerated, the size distribution is still uniform. The clear lattice fringe (Fig. 3a) and distinct SAED pattern (Fig. 2b) indicates the crystalline nature of the material. The lattice fringes can be resolved for the (111) and (1̄11) planes with measured interplanar spacings of 2.35 and 2.53 Å respectively. These values fit well with those of the pure CuO (JCPDS, file no. 48-1548). The SEM image obtained for CuO-A (Fig. 2d) displays aggregated structures of the oxide grains. It is believed that self-aggregation of the primary oxide nanoparticles (8 nm) leads to the formation of these micron-sized aggregates.

**Fig. 4:**
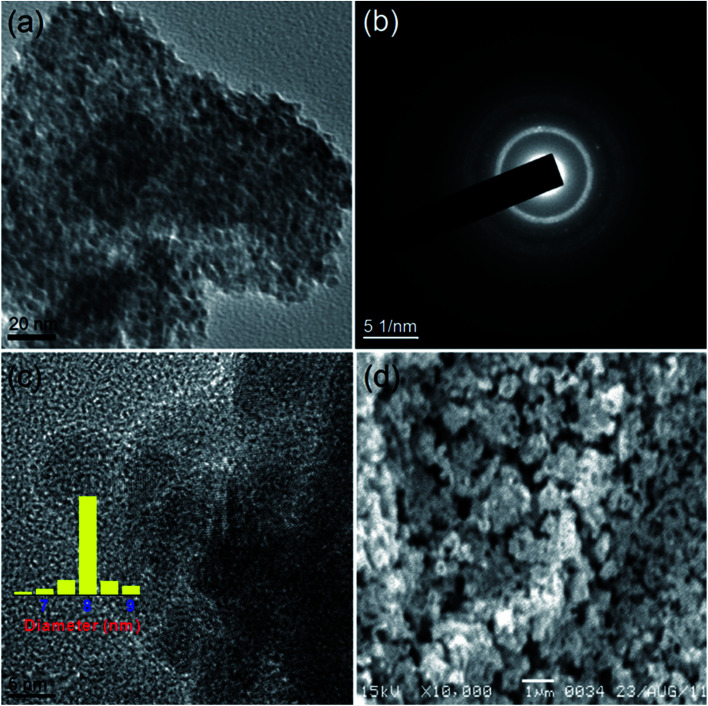
(a) TEM image, (b) SAED pattern, (c) HRTEM image and (d) SEM image of CuO-B.

Fig. 4 portrays the TEM and SEM images of CuO-B. Although, the TEM image displays the highly agglomerated particles, the HRTEM images show that the oxide grains are spherical in shape with an average diameter of 7 nm. The (111) and (1̄11) reflection planes can be identified in the lattice fringe (Fig. 3b) with measured spacings of 2.36 and 2.54 Å respectively. These two values are in good agreement with those of the phase-pure CuO (JCPDS, ref. no. 1-80-1916). The SEM image (Fig. 4d) shows that the oxide particles are highly agglomerated and look like porous aggregates. It can again be proposed that self-assembly of the primary particles leads to the formation of the observed aggregated structures. The average particle sizes of both the samples *viz.* CuO-A and CuO-B are almost same indicating that the added NaCl has no significant effect on the particle size.

**Fig. 5:**
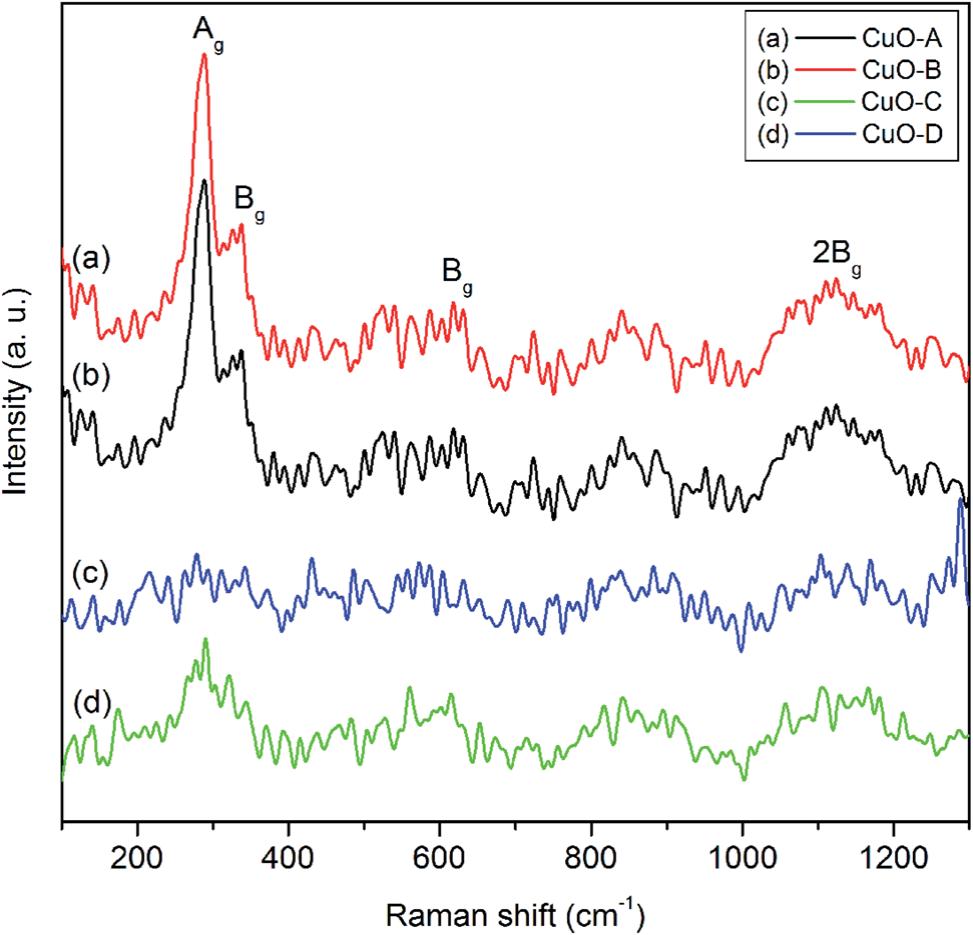
Raman spectra of the as-synthesized CuO samples.

Raman spectroscopy is a very sensitive tool to study the local atomic arrangements and vibrations of the materials which has been widely used to investigate microstructure of the nanoscale materials.^38^ CuO belongs to the *C*2/*c* space group with two molecules per primitive cell. There are 12 zone-center optical phonon modes such as*Γ*_RA_ = (4A_u_ + 5B_u_ + A_g_ + 2B_g_) in the phase-pure copper oxide. Amongst these 12 modes, (A_u_ + 2B_u_) are known as the three acoustic modes, (3A_u_ + 3B_g_) are the six infrared-active modes and (A_g_ + 2B_g_) are the three Raman-active modes.^8^Fig. 5 shows the three one-phonon peaks at 287, 338 and 614 cm^−1^ for both the CuO-A and CuO-B. The peak at 282 cm^−1^ can be assigned to A_g_ mode while the two peaks located at 338 cm^−1^ and 614 cm^−1^ can be assigned to the B_g_ modes.^9^ In addition to these three main one-phonon Raman scattering modes, another slightly broadened peak centered around 1130 cm^−1^ can be observed in the Raman spectra of all the oxide samples. This can unambiguously be ascribed to the multi-phonon (MP) transition (B_g_).^10^ It is known that the MP band arises due to anharmonic coupling of the phonons in polar solid. Intensity of the MP band is much lower than those of the one-phonon bands because intensity of the Raman peaks is related to the electron–phonon interaction.

The less intense and broaden peaks in both the Raman spectra of CuO-C and CuO-B suggest the non-crystalline or amorphous nature of the materials. This is consistent with the XRD study. A characteristic feature of the amorphous phase is the absence of long-range correlations that exists in the crystalline phase. Moreover, the short-range structural order present in the amorphous phase may be significantly different from that of the crystalline phase. Literature reports that in an amorphous material relaxation or breakdown of the Raman selection rules often arises due to the lack of long-range order and also due to the occurrence of disorder.^39^ This effect is believed to be responsible for the occurrence of a broad range of allowed scattering processes.^39^ The ultimate consequence is that broadening of the Raman lines takes place with decreasing crystal size due to the phonon confinement. This is why the amorphous materials show a strong broadening of the Raman modes. It is evident from the spectra that intensities of the Raman peaks increase upon calcination of the non-crystalline oxide samples which is in good agreement with the earlier findings.^40^

**Fig. 6:**
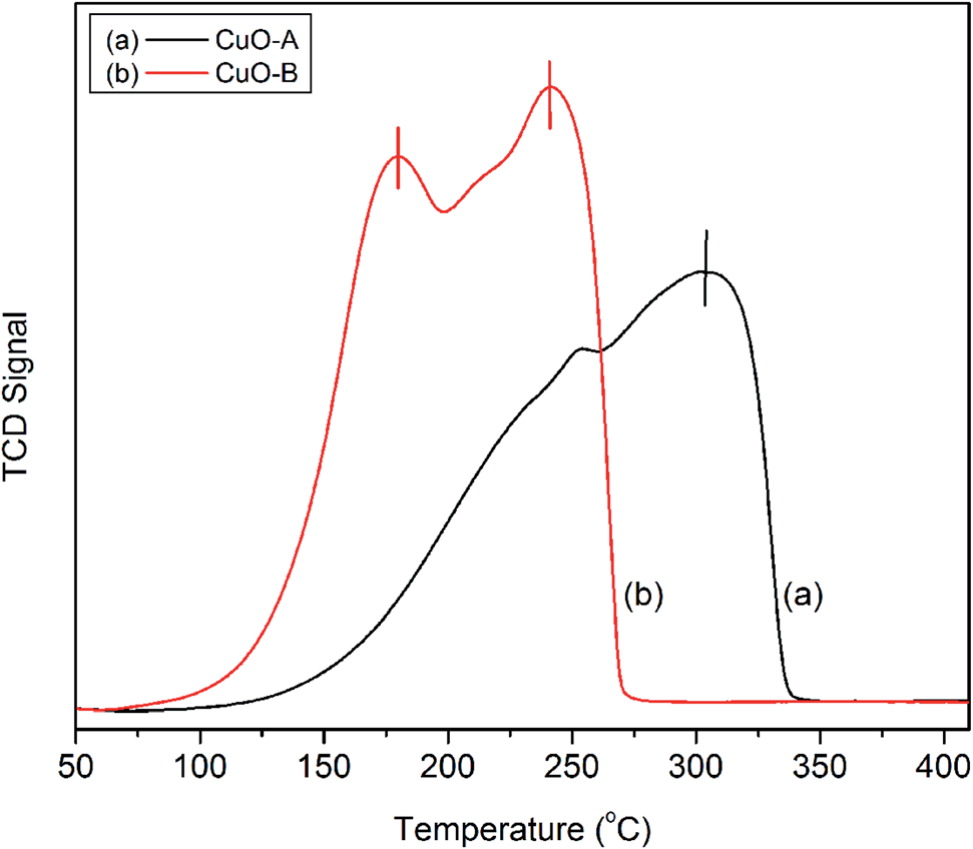
H_2_-TPR profiles of the crystallineCuO samples.

The H_2_-TPR profiles of the crystalline oxide samples are shown in Fig. 6. CuO-A exhibits a single major reduction peak around 302 °C. This is consistent with the literature reports which suggest that pure CuO shows only one reductive peak around 300 °C.^41^ On the other hand; the TPR profile of CuO-B exhibits two distinct peaks at 181 and 242 °C respectively. Fierro*et al.* have reported that TPR profile of an oxide material is markedly affected by an incorrect combination of initial amount of reducible species, total flow rate, initial hydrogen concentration and heating rate.^42^ They observed both single and double reductive peaks for CuO under different experimental conditions. In the present study, the two reductive peaks are believed to arise from the incorrect experimental condition.

### 3.2 Catalytic performance of CuO nanoparticles

Catalytic performances of the four oxide samples prepared as described above were investigated in the epoxidation of styrene under identical conditions to observe that the two uncalcined samples *viz.* CuO-C and CuO-D were relatively poor candidates as catalysts for the chosen reaction. CuO-C gives 67% styrene conversion with 73% epoxide selectivity, while CuO-D gives 61% styrene conversion with 69% epoxide selectivity. Therefore, further catalytic studies were employing only the calcined samples, *i.e.* CuO-A and CuO-B in the epoxidation reaction using aqueous (70%) *tert*-butyl hydroperoxide (TBHP) as the oxidant under heterogeneous condition. Results of the epoxidation reactions over our catalysts are presented in Table 2. It shows that both the catalysts exhibit better catalytic performance than the commercial CuO which gives only about 30% styrene conversion with barely 35% epoxide selectivity after 6 h of reaction. It further shows that CuO-A is catalytically more active than CuO-B giving complete styrene conversion with 95% epoxide selectivity. While CuO-B gives lower styrene conversion (78%) with almost same epoxide selectivity (92%) compared to CuO-A, under the identical condition.

**Table 2:** Catalytic epoxidation of styrene at different times over CuO catalyst^a^

Time (h)	Styrene conversion (%)	Selectively (%)		
		Styrene oxide	Benzaldehyde	Other products^c^
0.3	19 (11)^b^	90 (90.7)	4.43 (5.8)	5.57 (3.5)
1	32 (17.8)^b^	91 (90.4)	2.56 (3.9)	6.4 (5.7)
2	44 (32)^b^	89 (92.8)	1.6 (1.7)	9.4 (5.5)
3	60 (44)^b^	91.1 (92)	1.4 (1.2)	7.5 (6.8)
4	80 (56)^b^	89.5 (92.1)	2.2 (1.1)	8.3 (6.8)
5	92 (65.3)^b^	90 (92.2)	3.2 (0.9)	4.8 (6.8)
6	100 (78.2)^b^	95 (91.7)	2.5 (2.8)	6.5 (5.5)

a 5 mmol of styrene, 14 mmol of TBHP (aqueous, 70 wt%) and 10 mg of catalyst (CuO-A) were stirred in 10 mL of acetonitrile under reflux (85 °C) for different length of times.

b Catalyst CuO-B was used instead of CuO-A under identical reaction condition.

c Other products = benzoic acid, phenyl acetic acid *etc.*

**Fig. 7:**
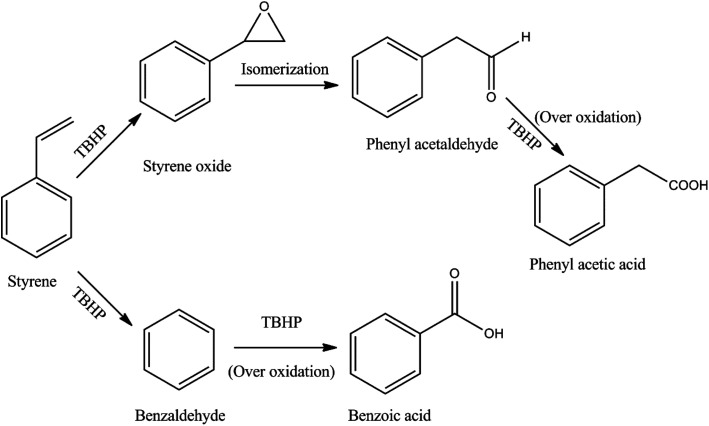
Reaction scheme for the epoxidation of styrene using TBHP.

The observed variation in the product selectivities indicates that two parallel consecutive reactions take place during the epoxidation of styrene using TBHP as the oxidant (Fig. 7). The lower selectivity for benzaldehyde compared to styrene oxide suggests that the TBHP causes a faster epoxidation than oxidation of styrene involving the elimination of CO_2_. However, as revealed by gas chromatography, the epoxidation product undergoes isomerization to an appreciable extent giving phenyl acetaldehyde as a byproduct which undergoes further oxidation to produce phenyl acetic acid in low concentration. Benzaldehyde also suffers from the over oxidation to its corresponding acid. An epoxidation reaction carried out under the typical condition without the CuO gives negligible styrene conversion suggesting that the presence of the catalyst during the reaction is essential and that neither TBHP nor air/O_2_ is capable of effecting the oxidative transformation.

Since, the average particle size of the two catalysts (CuO-A and CuO-B) is nearly the same, it is reasonable to assume that the significant difference in substrate conversion is not due to the effect of size or surface area the catalysts. Thus, the variation observed in the catalytic performances must be ascribed to other factors. It is believed that the higher catalytic activity of CuO-A compared to CuO-B is due to the greater abundance of surface defects of the oxide nanoparticles which were protected by the added NaCl during growth of the nanocatalyst particles. It was suggested earlier that significant reduction of surface defects could take place during the crystallite growth and Ostwald ripening in a coprecipitational synthesis of nanomaterials.^43^ Yuan *et al.* reported that in a reaction involving an occlusional coprecipitation, surface defects of CuO nanoparticles could be protected by NaCl formed *in situ*.^12^ Thus, it is believed that NaCl becomes adsorbed and deposited on the surface of the freshly formed CuO nanoparticles forming a salt sheath over the nanoparticles protecting the surface defects. The as-formed NaCl sheath actively retards the Ostwald ripening of CuO nanoparticles. The sheath-like salt layer behaves much like protective groups in organic synthesis and can protect surface defects by preventing the mass transference. The abundant surface defects can be recovered by removing the salt shield *via* washing with water. In present study, it is believed that the abundant surface defects are responsible for the greater selectivity of catalyst CuO-A compared to CuO-B. However, to completely understand how the added NaCl actually protects the surface defects of the nanoparticles, further work will be required.

Recently, Jia *et al.* have reported the synthesis of LDH-supported CuO nanorods of length 10–25 nm and diameter ∼3.6 nm which exhibit excellent catalytic performance giving 96% styrene conversion with 81% styrene oxide selectively at the end of 10 h catalytic epoxidation.^35^ In another recent instance, Hu *et al.* have reported the preparation of nanoscale CuO dispersed on CoAl-HT which shows 99.5% styrene conversion with 72% selectivity after 6 h of the catalytic reaction.^34^ In both the cases the catalytic reactions are carried out using TBHP as the oxidant. In our earlier work, we have observed73% styrene conversion with 77% epoxide selectivity over nanoscale α-Fe_2_O_3_ catalyst using TBHP as the oxidant after 6 h of epoxidation reaction.^44^ A comparison of our present work with these recent works on the epoxidation of styrene carried out almost under similar conditions suggests that the present works seems to be superior in all aspects including reaction times, conversion and selectivity.

To study the influences of different reaction parameters on the conversion and selectivity, several reactions were carried out using the superior catalyst CuO-A under the typical condition. When the reaction was carried out with a reduced amount of catalyst (5 mg), 84% styrene conversion with 91% epoxide selectivity was observed. Thus, a decrease in the amount of catalyst does not affect the epoxide selectivity but slightly decreases the styrene conversion. Both styrene conversion and styrene oxide selectivity were found to decline to 87% and 88% respectively on decreasing the amount of the oxidant to 10 mmol.

**Fig. 8:**
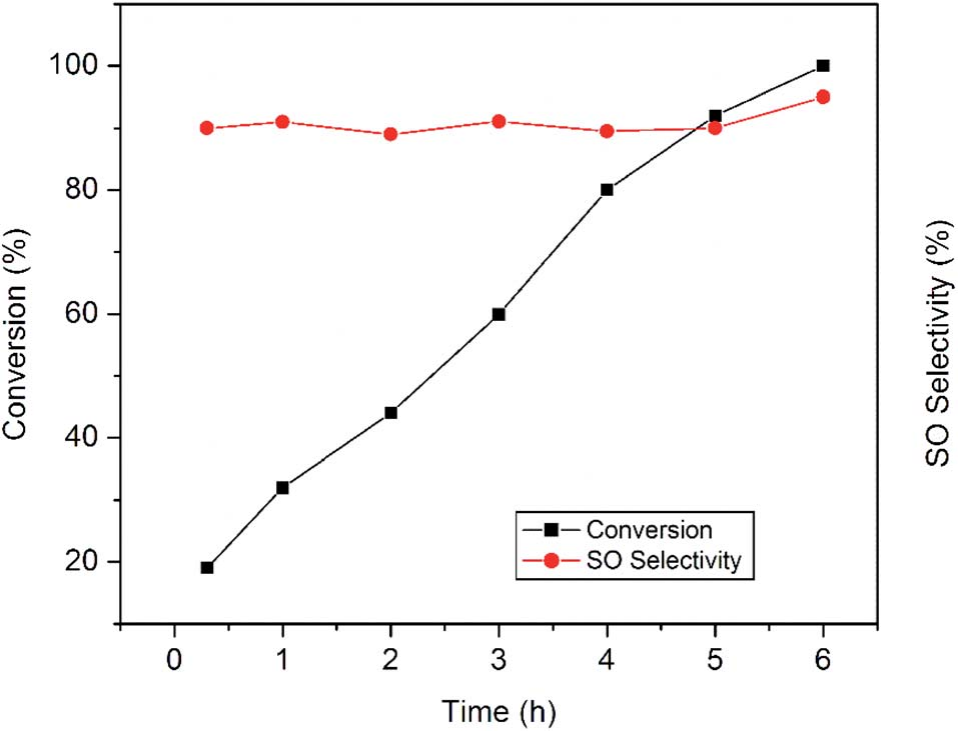
Influence of reaction time on the epoxidation of styrene over the CuO-A catalyst.

The plots in Fig. 8 clearly display the influence of reaction time on the epoxidation reaction. It was proposed that during epoxidation the side reaction involving oxidative cleavage of alkenes often becomes faster than the epoxidation leading to several undesired products.^11^ In the present work, active sites responsible for the side reactions are believed to be deactivated rather quickly and remain so throughout the reaction which is consistent with earlier findings.^11^ That is why, the production of benzaldehyde decreases rapidly to a minimum value and the epoxide selectivity remains almost constant. Prolonged run of the styrene epoxidation generally leads to the isomerization of styrene oxide into phenyl acetaldehyde which adversely affects the epoxide selectivity.^28^ But in our case, no significant isomerization has been observed. In the present study, the absence of side reactions and isomerization allows prolonging the epoxidation reaction which ultimately results in the complete styrene conversion with excellent epoxide selectivity.

**Fig. 9:**
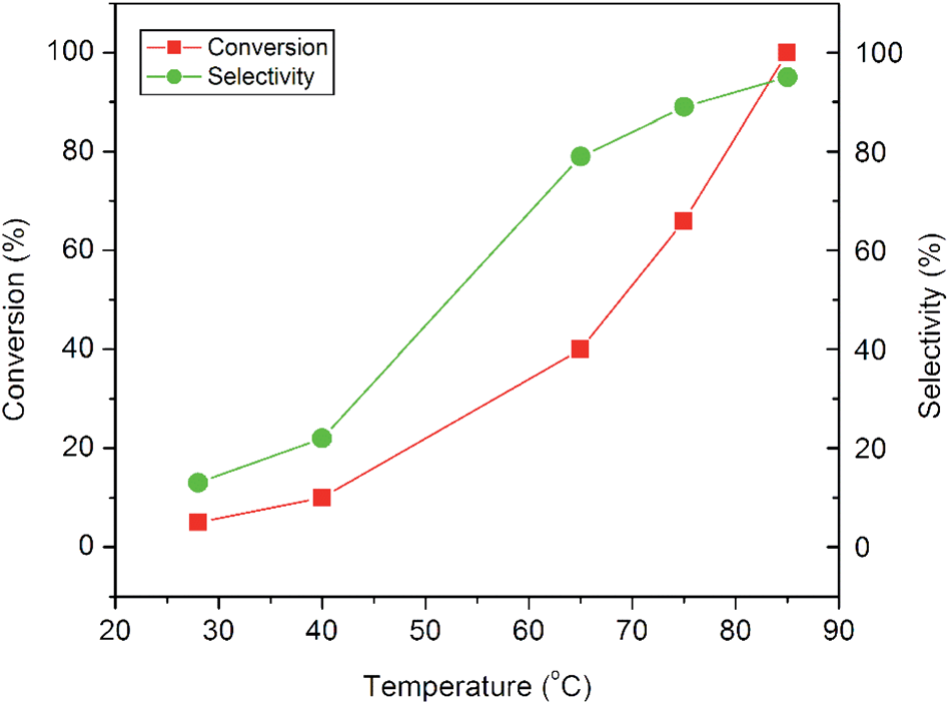
Influence of reaction temperature on the epoxidation of styrene over the CuO-A catalyst.

The plots in Fig. 9 show the influence of reaction temperature on the conversion and product selectivity in the epoxidation reaction. A gradual increase both in styrene conversion and in epoxide selectivity can be observed with increasing reaction temperature. As the reaction temperature is raised to 85 °C, the styrene conversion increases from 5 to 100% and at the same time the epoxide selectivity also increases from 13 to 95%. The observed increase both in conversion and selectivity may be due to the overall activation of the catalyst and the synchronous passivation of those catalytic sites which are responsible for the undesired oxidative cleavage.^11^

**Fig. 10:**
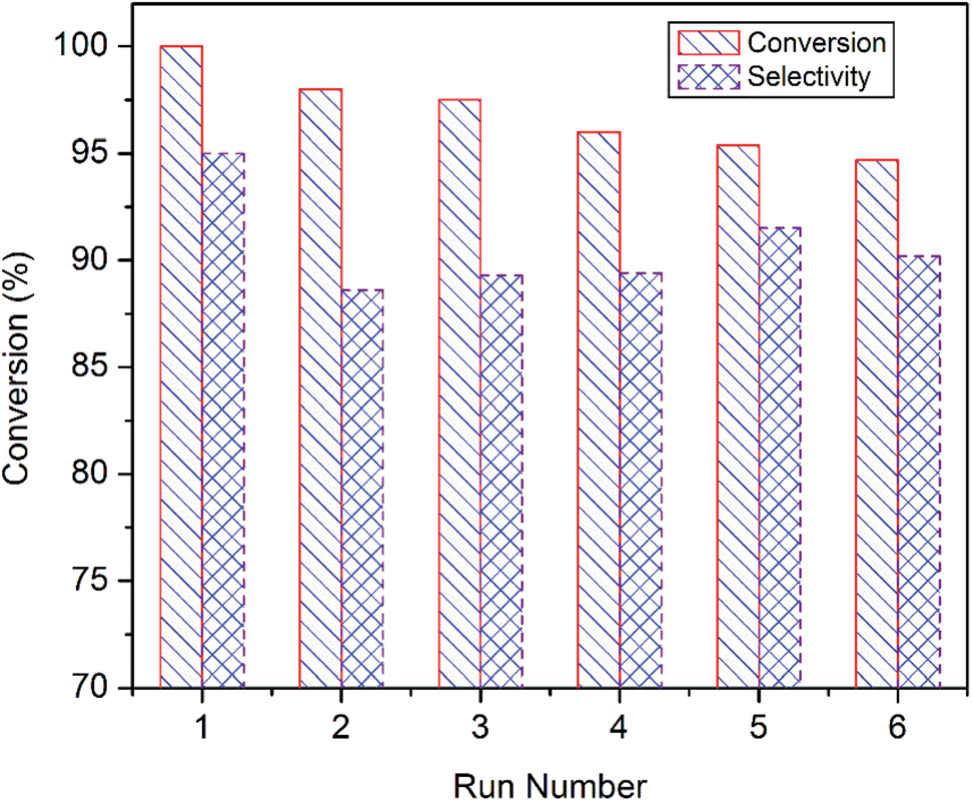
Recycling studies of CuO-A in the epoxidation reaction of styrene.

Reusability of the superior catalyst *i.e.* CuO-A was tested at 85 °C for 6 h (Fig. 10). After each run, the catalyst was recovered from the reaction mixture by means of centrifugation and washed several times with water and finally once with acetonitrile. The catalyst was dried at 60 °C for 6 h and then reused for the next run under the typical condition. It shows that the styrene conversion gradually decreases to 95% with successive reuse while the epoxide selectivity remains unaltered. Thus, the reusability experiments suggest that CuO-A can be reused at least for six successive runs without any significant fall in the catalytic performance. Further study will be needed to investigate whether any significant change takes place in the catalyst composition or not.

### 3.3 Electrochemical studies

**Fig. 11:**
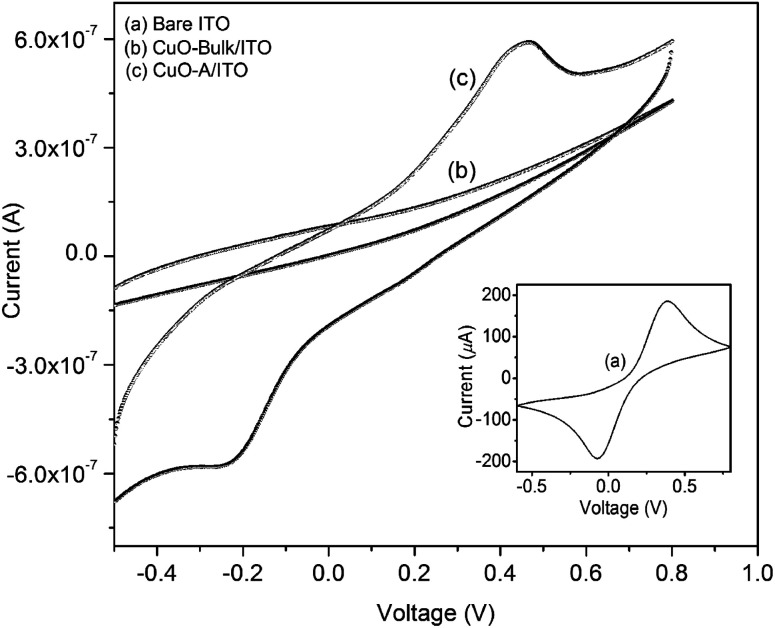
Cyclic voltammograms of the ITO supported CuO nanoparticles and bulk CuO particles. Inset shows the CV of bare ITO.

The cyclic voltammetry (CV) technique has been used to study the electrochemical behavior of the synthesized CuO nanoparticles and in all cases [Fe(CN)_6_]^3−/4−^ acts as redox couple. Fig. 11 displays the CV of bare ITO electrode (curve a), CuO-bulk/ITO (curve b) and CuO-A/ITO electrode (curve c). Inset shows a well-defined redox couple of [Fe(CN)_6_]^3−/4−^ for bare ITO electrode. The anodic current is as high as 186 μA which is due to the conductive nature of ITO. In case of CuO-bulk/ITO electrode, no redox peak of [Fe(CN)_6_]^3−/4−^ species is observed leading to the conclusion that an obstructed oxidation/reduction process results in the hindrance of ions towards the electrode. For CuO-A/ITO electrode, a well-defined oxidation/reduction process can be observed. This is due to the fact that CuO material in the nanometer dimension can help to mediate the electrons toward the electrode. The magnitude of peak current of the CuO-A/ITO electrode is decreased compared to that of the bare ITO electrode which can be attributed to the semiconducting nature of CuO nanoparticles. Ratio of the anodic peak current (*i*_p_: 0.6 μA) to the cathodic peak current (*i*_c_: 0.58 μA) is found to be ∼1.03 indicating a quasi-reversible and diffusion controlled process. Thus, the synthesized CuO-A nanoparticles exhibit a typical electrochemical behavior compared to the commercial bulk CuO particles.

Higher area of the oxidation/reduction peak for the synthesized nanoscale oxide, CuO-A indicates its smaller crystallite size or larger surface area than that of the commercial bulk counterpart which has relatively smaller redox peak area.^45^ Thus, CV investigation can be correlated to excellent catalytic performance of the nanoscale CuO-A on the basis of larger surface area of the oxide. Clearly, further work will be necessary to establish a direct relationship between the electrochemical behavior and catalytic performance of these newly synthesized oxide samples.

## 4 Conclusions

Ultra-small CuO particles with diameters of 7–8 nm have successfully been synthesized *via* a precipitation–calcination method using copper(ii) isonicotinatetetrahydrate as the precursor. The synthesized nanoscale CuO samples have shown excellent catalytic performance in the epoxidation of styrene using TBHP under heterogeneous condition. The abundant surface defects of the CuO nanoparticles, protected by the added NaCl during the synthesis, are believed to impart greater epoxide selectivity to the oxide catalyst. The catalyst can be recycled for several runs without any significant loss of both the activity and selectivity.

## Conflicts of interest

There are no conflicts to declare.

## Supplementary Material

## References

[cit1] Chen C., Cao Z., Zhang X., Li Y., Yuand L., Jiang X. (2020). Chin. J. Chem..

[cit2] Cao Z., Deng X., Chen C., Liu Y., Yu L., Jiang X. (2021). React. Chem. Eng..

[cit3] Ying C., Xiaobi J., Lei Y. (2020). Chin. J. Org. Chem..

[cit4] Wen X. G., Xie Y. T., Choi C. L., Wan K. C., Li X. Y., Yang S. H. (2005). Langmuir.

[cit5] Cao M. H., Hu C. W., Wang Y. H., Guo Y. H., Guo C. X., Wang E. B. (2003). Chem. Commun..

[cit6] Lu C. H., Qi L. M., Yang J. H., Zhang D. Y., Wu N. Z., Ma J. M. (2004). J. Phys. Chem. B.

[cit7] Liu Y., Chu Y., Li M. Y., Dong L. H. (2006). J. Mater. Chem..

[cit8] Gao D., Yang G., Li J., Zhang J., Zhang J., Xue D. (2010). J. Phys. Chem. C.

[cit9] Wang X., Xi G., Xiong S., Liu Y., Xi B., Yu W., Qian Y. (2007). Cryst. Growth Des..

[cit10] Singh D. P., Ojh A. K., Srivastava O. N. (2009). J. Phys. Chem. C.

[cit11] Chen C., Qu J., Cao C., Niu F., Song W. (2011). J. Mater. Chem..

[cit12] Zhang H., Cao J.-L., Shao G.-S., Yuan Z.-Y. (2009). J. Mater. Chem..

[cit13] Sukumar S., Rudrasenan A., Nambiar D. P. (2020). ACS Omega.

[cit14] Guzman M., Arcos M., Dille J., Rousse C., Godet S., Malet L. (2021). ACS Omega.

[cit15] Akintelu S. A., Folorunso A. S., Folorunso F. A., Oyebamiji A. K. (2020). Heliyon.

[cit16] Zedan A. F., Mohamed A. T., El-Shall M. S., AlQaradawi S. Y., AlJabera A. S. (2018). RSC Adv..

[cit17] Khaldari I., Naghavi M. R., Motamedi E. (2021). RSC Adv..

[cit18] Ghosh M. K., Sahu S., Gupta I., Ghora T. K. (2020). RSC Adv..

[cit19] Chowdhury R., Khan A., Rashid H. (2020). RSC Adv..

[cit20] Moamen S., El-Sayed M. Y., Adam A. M. A. (2013). J. Mol. Structure.

[cit21] Premkumar T., Geckeler K. E. (2006). J. Phys. Chem. Solids.

[cit22] Swern D. , Organic Peroxide, Wiley-Interscience, New York, 2nd edn, 1971, vol. 2

[cit23] Yin D., Qin L., Liu J., Li C., Jin Y. (2005). J. Mol. Catal. A: Chem..

[cit24] Ghosh R., Shen X., Villegas J. C., Ding Y., Malinger K., Suib S. L. (2006). J. Phys. Chem. B.

[cit25] Patil N. S., Uphade B. S., Jana P., Bhargava S. K., Choudhary V. R. (2004). J. Catal..

[cit26] Patil N. S., Uphade B. S., McCulloh D. G., Bhargava S. K., Choudhary V. R. (2004). Catal. Commun..

[cit27] Patil N. S., Uphade B. S., Jana P., Bhargava S. K., Choudhary V. R. (2004). Chem. Lett..

[cit28] Choudhary V. R., Jha R., Chaudhari N. K., Jana P. (2007). Catal. Commun..

[cit29] Zhang Y., Li H., Zhang L., Gao R., Dai W.-L. (2019). Chem. Eng..

[cit30] Guo S., Wang H., Tricard S., Zheng P., Sun A., Fang J., Zhao J. (2020). Ind. Eng. Chem. Res..

[cit31] Liang Y., Yi C., Tricard S., Fang J., Zhao J., Shen W. (2015). RSC Adv..

[cit32] Sun J., Kan Q., Li Z., Yu G., Liu H., Yang X., Huob Q., Guan J. (2014). RSC Adv..

[cit33] Sadasivan R., Patel A. (2019). RSC Adv..

[cit34] Hu R., Yang P., Pan Y., Li Y., He Y., Feng J., Li D. (2017). Dalton Trans..

[cit35] Jia W., Liu Y., Hu P., Yu R., Wang Y., Ma L., Wang D., Li Y. (2015). Chem. Commun..

[cit36] Das B. K., Bora S. J., Chakrabortty M., Kalita L., Chakrabarty R., Barman R. K. (2006). J. Chem. Sci..

[cit37] Terry K. W., Lugmair C. G., Gantzel P. K., Tilley T. D. (1996). Chem. Mater..

[cit38] Zi J., Buscher H., Falter C., Ludwig W., Zhang K., Xie X. (1996). Appl. Phys. Lett..

[cit39] Bersani D., Lottici P. P., Montenero A. (1999). J. Raman Spectrosc..

[cit40] Lai W. H., Teoh L. G., Su Y. H., Shieh J., Hon M. H. (2007). J. Am. Ceram. Soc..

[cit41] Zeng S. H., Liu Y., Wang Q. Y. (2007). Catal. Lett..

[cit42] Fierro G., Lojacono M., Inversi M., Porta P., Lavecchia R., Cioci F. (1994). J. Catal..

[cit43] Cushing B. L., Kolesnichenko V. L., O'Connor C. J. (2004). Chem. Rev..

[cit44] Bepari R. A., Bharali P., Das B. K. (2017). J. Saudi Chem. Soc..

[cit45] Jha A., Rode C. V. (2013). New J. Chem..

